# An analysis of virtual triage utilization by pregnant women prior to and during the COVID-19 pandemic

**DOI:** 10.3389/fgwh.2024.1423993

**Published:** 2024-10-31

**Authors:** Jakub Jaszczak, George A. Gellert, Gabriel L. Gellert, Aleksandra Suwińska

**Affiliations:** ^1^ Infermedica, Wroclaw, Poland; ^2^ Infermedica, San Antonio, CA, United States

**Keywords:** virtual triage, symptom checker, maternal care, telemedicine, pregnancy care, respiratory symptoms, digital triage, COVID-19

## Abstract

**Objective:**

This analysis describes the use patterns of web-based virtual triage (VT) by pregnant patients before and during the first two years of the COVID-19 pandemic, and how the pandemic influenced frequency of VT use, nature of symptoms reported, and the associated implications for maternal healthcare delivery.

**Methods:**

An online survey of 36,910 patients who reported pregnancy was completed between January 1, 2019 and June 30, 2022. The data were segmented into six month periods to allow comparative analyses of usage frequency and changes in initial complaints over the study period, with particular emphasis on the early months of the COVID-19 pandemic. Descriptive statistics and trend analyses were used to identify significant shifts in symptom reporting and user demographics.

**Results:**

A marked increase in the utilization of VT by pregnant women during the pandemic occurred. The percentage of pregnant users grew from 0.32% in the first half of 2019 to 0.85% in late 2021, with the greatest rise (213%) in the first six months of 2020. The most common symptoms reported were abdominal pain, headache, nausea, back pain, fatigue and cough. Pre-pandemic, VT use focused on prospective mothers learning about the potential causes of typical symptoms occurring during pregnancy, but during the pandemic there was a substantial increase in reporting symptoms associated with acute respiratory infections such as cough, nasal congestion, and dyspnea.

**Conclusions:**

The COVID-19 pandemic significantly influenced the use of VT by pregnant women, with a shift towards addressing concerns related to respiratory symptoms and potential COVID-19 exposure. These findings underline the significant role of digital health tools in maintaining access to health information during times of crisis and highlight the evolving needs of pregnant patients in such settings.

## Introduction

On March 11, 2020 the World Health Organization (WHO) declared that the spread of COVID-19 constituted a global pandemic. Healthcare delivery personnel were re-assigned to provide treatment to COVID-19 patients. Access to routine healthcare became severely limited. Initial uncertainty and miscommunication about COVID-19 preventive measures led to fear of accessing care (1). On March 13, COVID-19 was declared a National Emergency in the US, leading to an expansion of telehealth services and wider use of digital health to protect patients and providers from infection (2). The expansion of telehealth was welcomed by many patients to avoid unnecessary visits to an emergency department, urgent care, or clinics (3, 4). Evidence demonstrates that telehealth and digital tools can improve care outcomes and reduce costs (5).

Maternal and pregnancy care presents particular challenges to virtual healthcare due to the need for recurrent medical evaluations, and increased risk of severe complications from possible COVID-19 infection during pregnancy (6, 7). The contraction of upper respiratory infections, such as COVID-19, during pregnancy can have adverse effects on mothers and newborns alike (8). Debolt et al. demonstrated that pregnant women who contracted COVID-19 had increased morbidity compared to nonpregnant controls (9). A systematic review found that pregnant women with influenza were at elevated risk of morbidity and death, while infants born to mothers with influenza infection had higher risk of preterm birth and/or low birthweight, each of which increases mortality risk (10). Furthermore, infection with influenza in infants under six months old results in the highest rates of hospitalization and death among all children (10).

WHO has recognized the value of telehealth for counseling and screening of pregnant patients, including checks for potential danger signs (1). However, risk related to unattended pregnancy and childbirth outweighs potential risks of coronavirus transmission among mothers and newborns (1). Evidence suggests that implementation of telehealth services in maternal and newborn healthcare may improve obstetric outcomes related to patient education (e.g., smoking cessation or continuation of breastfeeding), and for acute interventions (monitoring of high-risk pregnancies, access to early abortion) (11). Concerns about contracting SARS-CoV-2 infection, resulting pandemic lockdowns, limited access to in-person healthcare, and increased fear and anxiety in pregnant patients during the pandemic were associated with higher odds of depressive episodes (12, 13). Telehealth can also serve to alleviate stresses and anxieties associated with exposure to COVID-19 (14). Patients seeking reassurance through teleconsultation have utilized virtual and digital health applications, such as virtual or online symptom triage, to check whether symptoms experienced are normal for a pregnant person (15).

This paper assessed the increased usage of virtual triage (VT) by pregnant patients before and at the beginning of the COVID-19 pandemic, and then over the ensuing two years. These analyses describe the most common patient inquiries, usage patterns, and patterns of medical guidance generated by the use of a leading virtual triage engine. The VT engine that collected the data for this analysis, was Symptomate from Infermedica. Symptomate is an artificial intelligence (AI) driven symptom checker or virtual triage and care referral engine available online at no cost; it is primarily different from other symptom checkers by its deployment of AI and a clinical database designed and continually updated by a team of over 50 licensed physicians.

## Methods

### Study objectives

The objective of this study was to identify and describe changes in the frequency of VT usage and patterns of clinical complaints reported by pregnant patients before the outbreak and during the first 30 months of the COVID-19 pandemic.

### Virtual triage technology and workflow

Symptomate is a standalone virtual triage engine or symptom checker from Infermedica designed for patient-users seeking online symptom triage. The VT engine estimates the probability of specific diseases based on information collected systematically from patient-users, and issues recommendations for further treatment or contact with a healthcare professional, or self-care and monitoring, as clinically appropriate and needed. The triage engine is available through the Infermedica website and as a mobile downloadable application from the Apple Store and Google Play.

**Figure 1:**
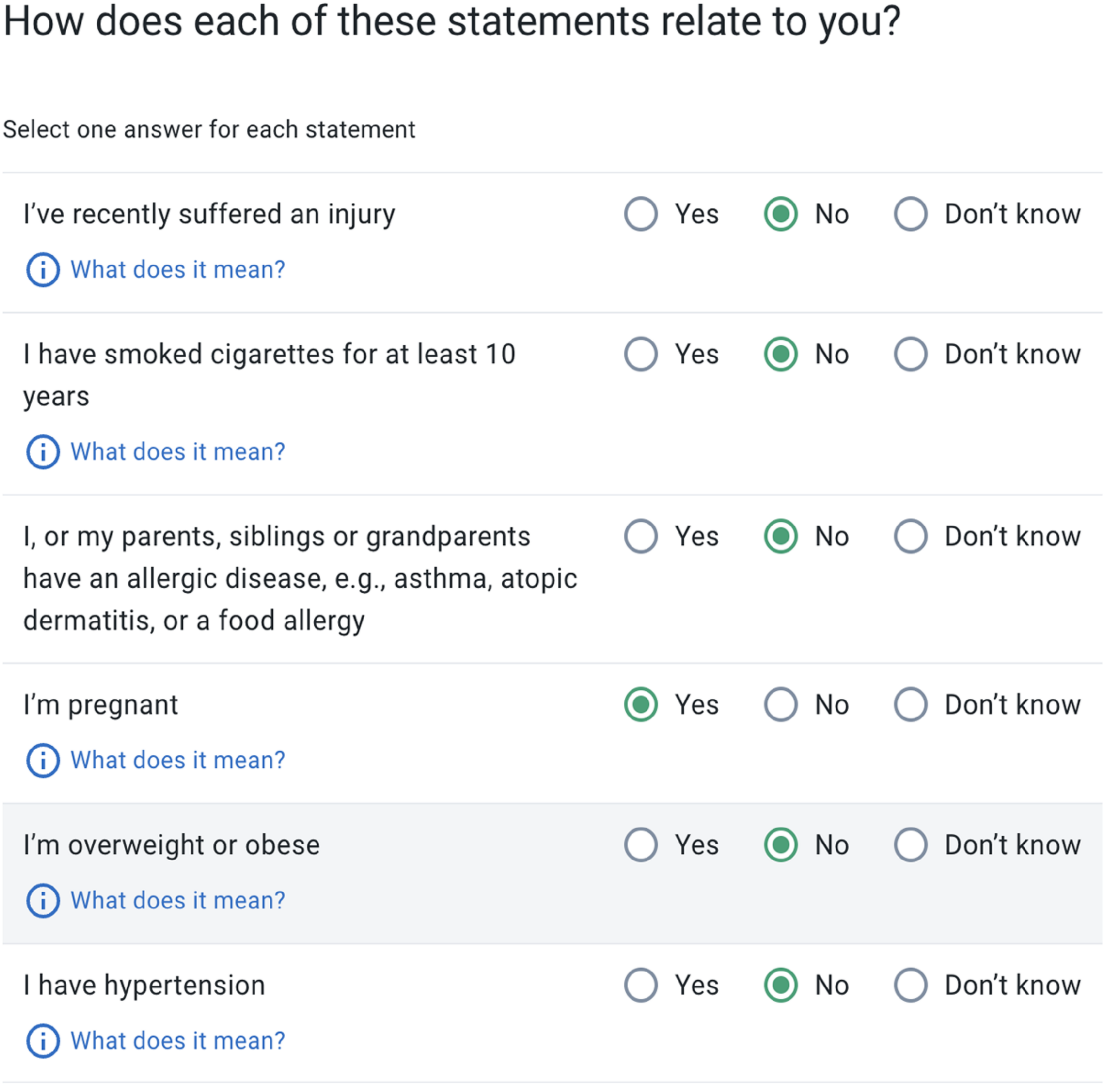
Virtual triage survey questions on health history and comorbidities.

In each VT session patient-users provide their gender and age, any risk factors, significant past medical history and comorbidities, place of residency, recent travel history and initial symptoms (Figure 1).

**Figure 2:**
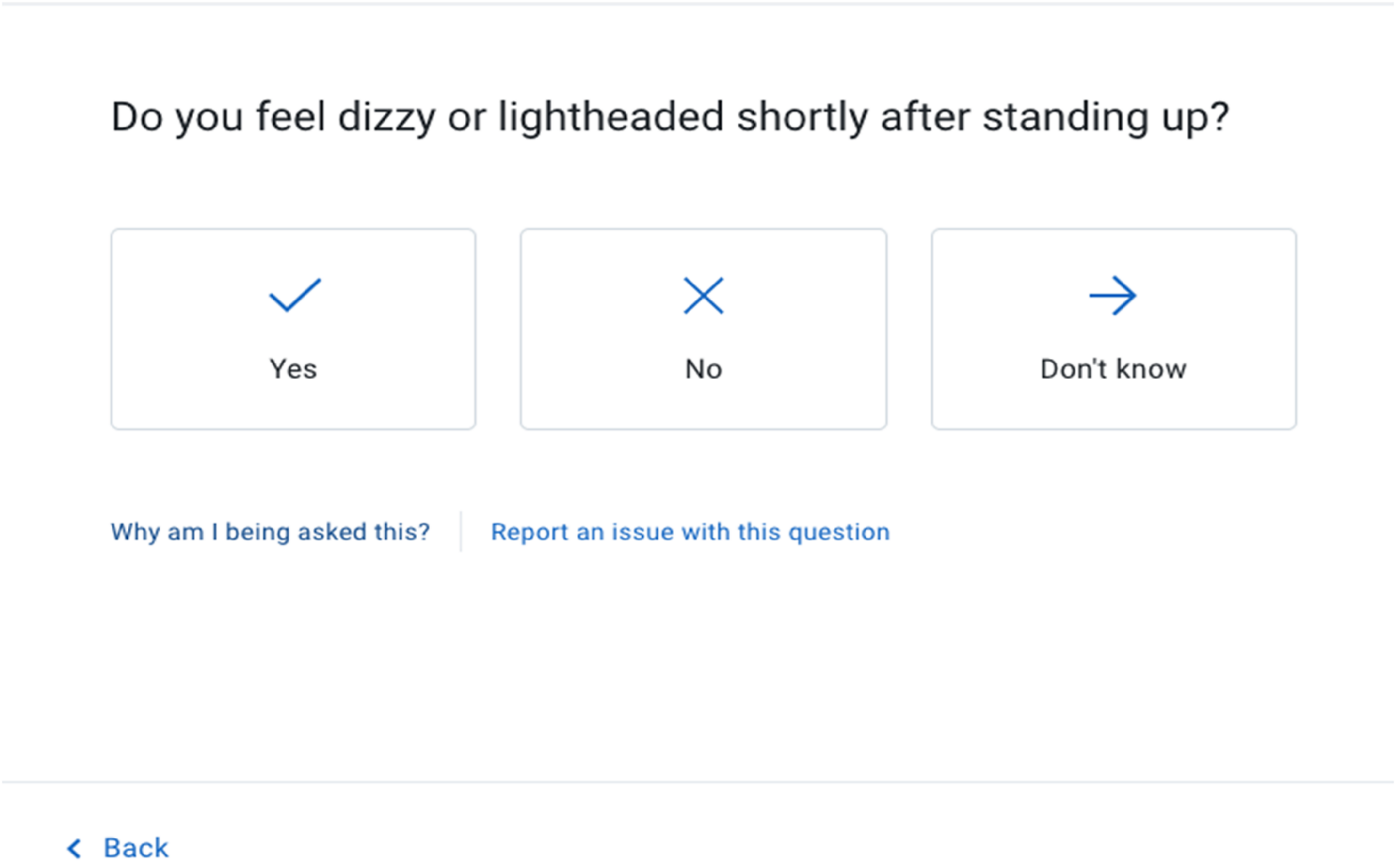
Sample virtual triage question about symptoms.

The VT engine then asks a series of about 15 questions about potential symptoms that, according to its algorithms run by artificial intelligence, are most related to potential causes of the patient's initial complaints (Figure 2). Finally, a list of most probable causes of the patient's symptoms is displayed, along with a care recommendation and pertinent patient education. Symptomate VT coverage of health issues encompasses over 1,700 symptoms (16). 300 risk factors and over 800 diseases (17, 18).

Patient-users convey their risk factors in two ways at the beginning of the interview - by reporting them along with their initial symptoms, or through a dedicated Risk Factor Screen that requires a response (yes/no/I don't know) to the most common demographically linked risk factors (typically between 4 and 10) according to patient-user age and sex. For female patient-users aged 18–44, pregnancy is always displayed on the Risk Factor Screen, and may be reported by any female aged 12–64.

### Data preparation and analysis

The dataset for this study included 4,926,587 VT encounters conducted via Symptomate between January 1, 2019, and June 30, 2022. From this dataset, we identified 36,910 encounters where users reported pregnancy. Due to technical limitations, in encounters from the first half of 2020 we included all pregnancies logged by patients, regardless of whether they reported it as an initial complaint or were asked about that during the interview. Data was divided into six month intervals to facilitate time-based analysis. Data cleaning removed inconsistent, incomplete or invalid entries. Criteria for removing VT interviews included: interviews that were incomplete; interviews where a user decided to go back and modify their responses; and interviews where the user did not report any symptom at the beginning of the interview

Symptoms reported by users were categorized into relevant medical categories to streamline analysis according to the medical meaning for the user—e.g., headache, nausea. The most common symptoms are listed in Table 1. Data was normalized to account for variations in the number of users across different periods. The primary analytic objective was to evaluate changes in the frequency and types of symptoms reported by pregnant users over the specified time periods. Analytical methods included: (1) descriptive statistics calculated to understand the distribution and central tendencies of the reported symptoms; (2) trend analysis of changes in symptom reporting over successive six month periods to identify significant increases or decreases in particular symptoms; (3) comparative analysis of the occurrence of each symptom across different periods and age groups, with notable changes highlighted. It is important to note that a diagnosis of COVID-19 was determined either from patient self-reporting or deduced by the artificial intelligence within the virtual triage engine; there was no laboratory confirmation of patients as positive for SARS-CoV-2 infection.

**Table 1:** Most common initial complaints reported by pregnant virtual triage users.

Symptom	Number of triage complaints	Triage episodes with pregnant users
Abdominal pain	11,845	32.1%
Headache	9,928	26.9%
Nausea	6,073	16.5%
Back pain	5,941	16.1%
Fatigue	5,252	14.2%
Cough	3,930	10.6%
Dizziness	3,430	9.3%
Pharyngeal pain	3,027	8.2%
Nasal congestion	2,821	7.6%
Vomiting	2,758	7.5%
Diarrhea	2,583	7.0%
Dyspnea	2,519	6.8%
Chest pain	2,510	6.8%
Fever	2,232	6.0%
Joint pain	1,916	5.2%
Nasal catarrh	1,884	5.1%
Edema	1,649	4.5%
Abnormal vaginal discharge	1,556	4.2%
Dermatological changes	1,547	4.2%
Bloating	1,514	4.1%

Statistical analysis was performed to quantify changes in symptom prevalence, including percentage change for each symptom across successive six month periods. For instance, from the first six months of 2020 to the second half of 2019, the reporting of cough increased by 199.0%, while nausea decreased by 28.9%.

## Results

### Patient-User demographics

The 36,910 patients who reported pregnancy were qualified for further analysis in the study. A majority of patients (67.6%) were aged between 18 and 29 years, and 31.8% of patient-users were aged 30–44 years.

### Patient-User language distribution

**Figure 3:**
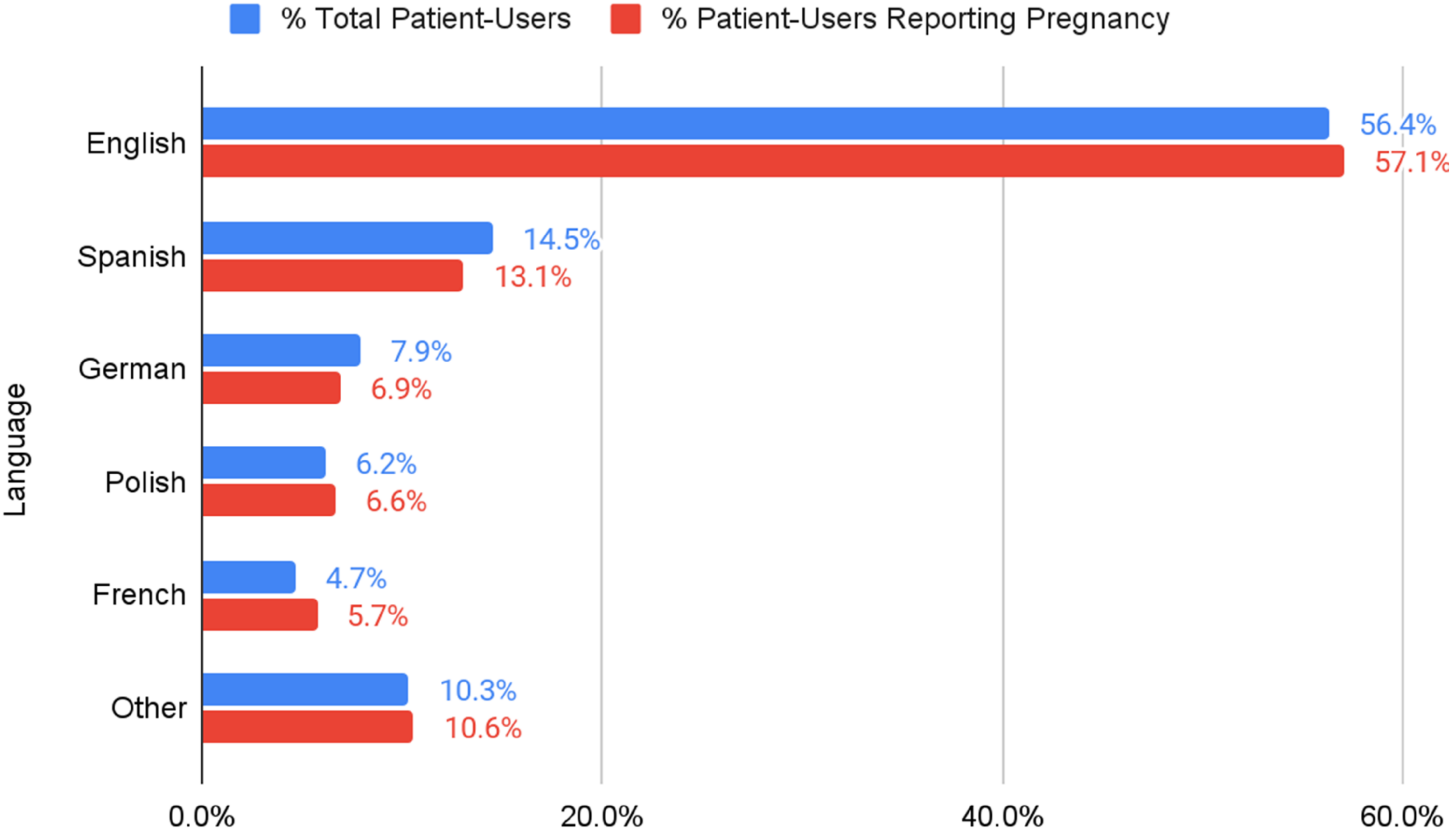
Interview language distribution of patient-users reporting pregnancy.

Users could choose from among 15 languages to complete the virtual triage interview. The majority of online interviews during the study period were conducted in English (57.1%), followed by Spanish (13.1%), German (6.9%), Polish (6.6%), and French (5.7%). Figure 3 depicts the percentage of total patient-users by language group and the percentage of patient-users that reported pregnancy for each language group.

### Virtual triage utilization by pregnant patient-users

During the 30-month period between January 1, 2019 and June 30, 2022 there were 4,926,587 triage encounters completed on Symptomate. Of these, 36,910 (0.75%) patient-users reported pregnancy at the beginning of their VT session.

### Frequency of virtual triage usage by study period

The number of encounters among pregnant patient-users per time period rose four-fold over the course of the study period, from 244,299 to 1,071,334 encounters (Table 2). Across the entire study period, the greatest increases in VT usage were observed during the second half of 2020, and the first half of 2021. These overall use patterns may correspond to increasing public anxiety concerning rising COVID-19 incidence, deployment of lockdown measures to reduce community transmission of the virus, and the emergence of new viral variants.

**Table 2:** Pregnant patient-users virtual triage encounters by six month intervals.

Six month interval*							
	Q1-2	Q3-4	Q1-2	Q-3-4	Q1-2	Q3-4	Q1-2
	2019	2019	2020	2020	2021	2021	2022
Total Number of Users	244,299	400,310	591,414	596,870	1,064,440	1,071,334	957,920
Change in relation to previous period	−	63.86%	47.74%	0.92%	78.34%	0.65%	−10.59%
Number of users reporting pregnancy	793	1,404	4,392	4,456	8,837	9,099	7,929
Change relative to previous period	−	77.05%	212.82%	1.46%	98.32%	2.96%	−12.86%
Percent reporting pregnancy among all users	0.32%	0.35%	0.74%	0.75%	0.83%	0.85%	0.83%

Note: Q refers to a quarter of the year.

**Figure 4:**
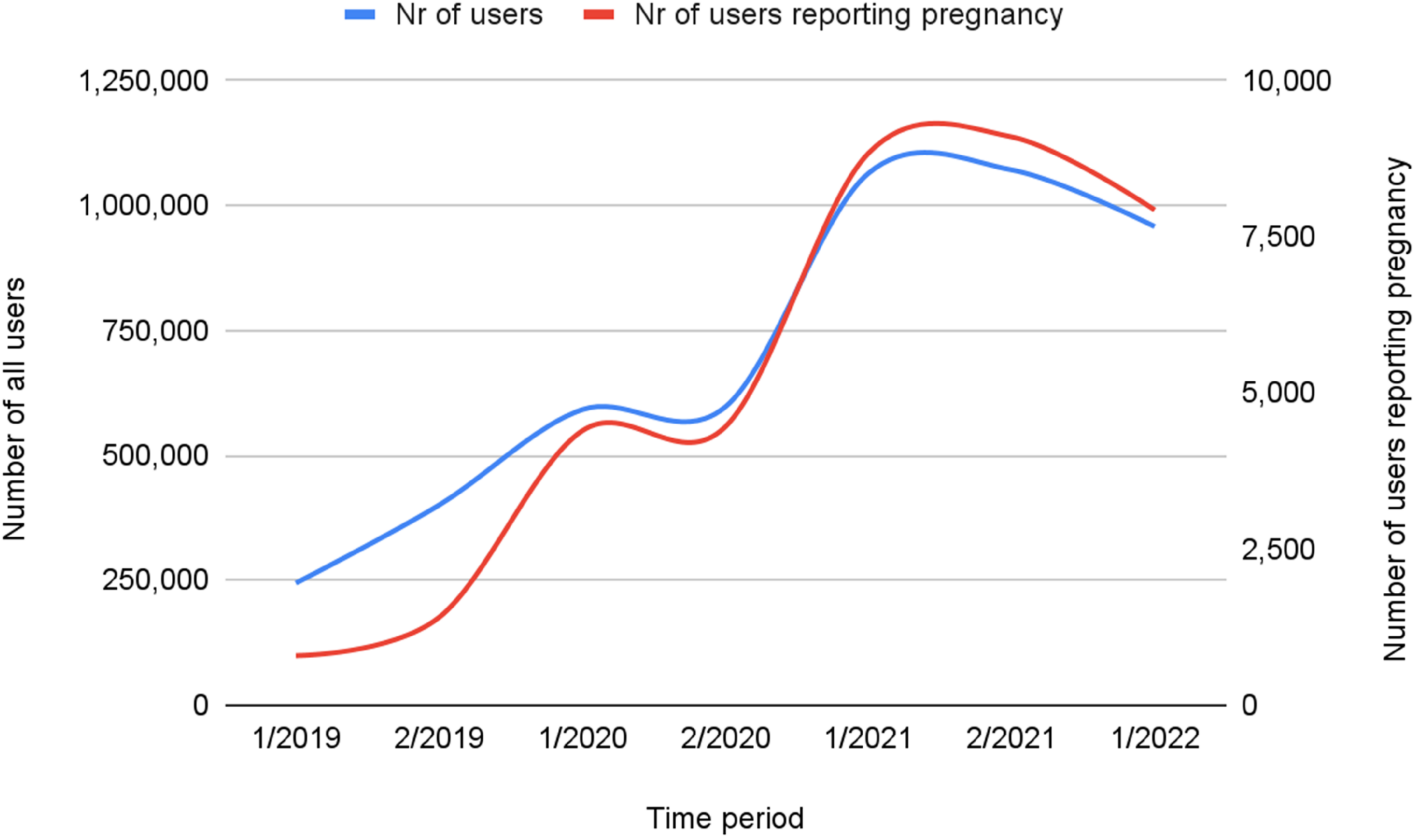
Change in number of patient-users reporting pregnancy Vs. all patient-users.

Figure 4 illustrates the change in number of patient-users reporting pregnancy vs. all virtual triage patient-users, which was greater than that observed for the overall population. The number of users reporting pregnancy over the study period increased tenfold, with the greatest increase, of over 200%, observed in the first half of 2020, as pandemic lockdowns were introduced. Despite the general increased use among all users during this period, the percentage of encounters in which a user reported pregnancy more than doubled, from 0.3% to 0.85%.

### Patient-Users gender and age breakdown

**Figure 5:**
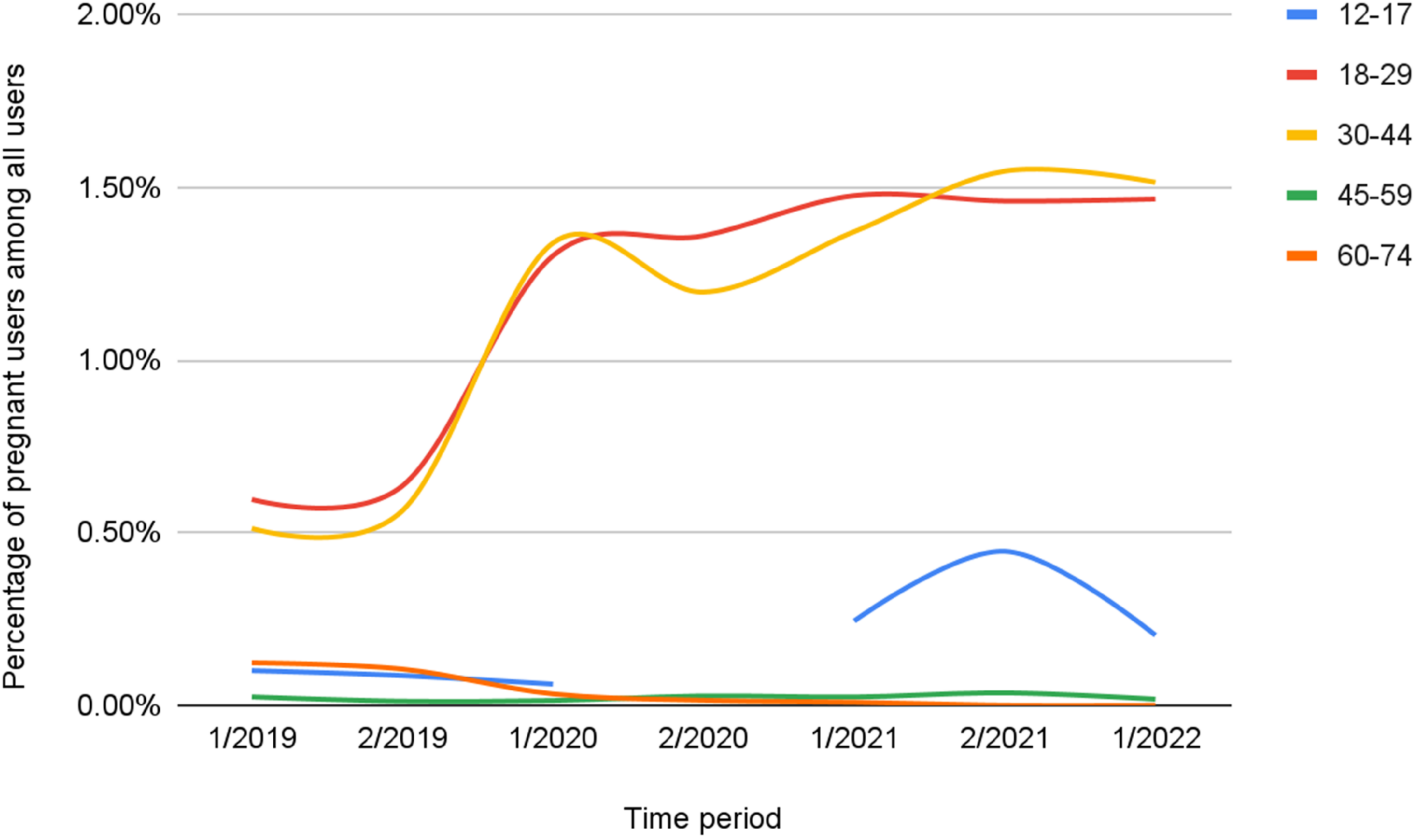
Percentage of users reporting pregnancy among all female virtual triage users by age.

Table 3 shows patient-users by age. The most common age group in which pregnancy was reported was between 18 and 29 years, followed by 30–44 years. The percentage change in pregnant users among all users, per time period, shows that those aged 18–44 years were the most common age stratum for pregnant users (Table 3), but also was the most rapid growth in number of users, especially during the first half of 2020 (Figure 5).

**Table 3:** Prevalence of reported pregnancy among female users in each age group.

Age	Number of female users	Number of pregnant users	Percentage of pregnant users among all female users
12–17	47,458	105	0.2%
18–29	1,880,195	24,956	1.3%
30–44	906,709	11,737	1.3%
45–59	378,594	89	0.02%
60–74	96,950	23	0.02%

Figure 5 conveys the percentage of users reporting pregnancy among all females by age stratum.

### Initial clinical complaints of pregnant users

Pregnant users reported fewer initial complaints than all virtual triage users (−24.7%). During the study period, 424 unique initial complaints were reported by pregnant users, but only 20 symptoms occurred in greater than 4.0% of pregnant user triage episodes, comprising 86.5% of all complaints among pregnant users (one user might have reported more than one initial complaint). More than 10.0% reported one of six symptoms, including abdominal pain, headache, nausea, back pain, fatigue, or cough.

### Changes in patient-user initial complaints over time

Table 4 reports change in frequency of commonly reported symptoms over time as a percentage of VT encounters per six month period.

**Table 4:** Most frequent initial symptoms by six month time periods.

Six month time period								
Symptom	All periods	Q1-2 2019	Q3-4 2019	Q1-2 2020	Q-3-4 2020	Q1-2 2021	Q3-4 2021	Q1-2 2022
Abdominal pain	32.1%	45.0%	42.2%	32.3%	33.2%	31.7%	29.8%	31.4%
Headache	26.9%	23.3%	21.6%	25.8%	26.8%	26.9%	28.4%	27.2%
Nausea	16.5%	21.4%	23.2%	16.5%	16.5%	16.4%	15.6%	15.7%
Back pain	16.1%	19.2%	19.7%	15.7%	16.0%	15.9%	15.9%	15.8%
Fatigue	14.2%	1.1%	7.3%	13.8%	14.0%	14.1%	15.6%	15.7%
Cough	10.6%	5.0%	3.6%	10.9%	9.1%	8.8%	14.2%	11.1%
Dizziness	9.3%	10.8%	13.5%	8.8%	9.9%	9.3%	9.0%	8.7%
Pharyngeal pain	8.2%	1.6%	2.8%	7.4%	7.4%	7.0%	11.3%	8.5%
Nasal congestion	7.6%	2.3%	2.6%	5.2%	7.2%	7.2%	11.0%	7.3%
Vomiting	7.5%	8.6%	9.0%	6.6%	6.4%	7.1%	7.7%	8.3%
Diarrhea	7.0%	8.2%	8.3%	6.2%	6.7%	6.7%	7.6%	7.0%
Dyspnea	6.8%	6.7%	5.6%	7.8%	7.9%	6.5%	6.9%	6.2%
Chest pain	6.8%	5.4%	3.9%	7.4%	7.2%	6.4%	7.2%	6.9%
Fever	6.0%	2.8%	5.8%	5.8%	5.3%	5.4%	6.8%	6.8%
Joint pain	5.2%	4.9%	4.7%	5.2%	5.4%	5.3%	5.0%	5.3%
Nasal catarrh	5.1%	1.1%	1.0%	3.7%	4.1%	4.5%	7.6%	5.4%
Edema	4.5%	6.2%	3.8%	5.0%	4.4%	5.2%	3.9%	4.0%
Abnormal vaginal discharge	4.2%	4.2%	7.0%	4.6%	4.4%	4.1%	3.5%	4.3%
Dermatological changes	4.2%	3.4%	2.8%	3.3%	4.1%	5.0%	4.0%	4.4%
Bloating	4.1%	6.6%	6.5%	4.4%	4.6%	4.0%	3.4%	3.9%

Note: Q refers to a quarter of the year.

Table 5 compares the prevalence of symptoms across six month time periods. Increases in prevalence of greater than 20.0% are noted in green and decreases of greater than 20.0% are red.

**Table 5:** Symptom prevalence changes among pregnant patient-users by six month period.

Symptom	Q3-4/2019 vs. 1-2/2019	Q1-2/2020 vs. Q3-4/2019	Q3-4/2020 vs. Q1-2/2020	Q1-2/2021 vs. Q3-4/2020	Q3-4/2021 vs. Q1-2/2021	Q1-2/2022 vs. Q3-4/2021
Abdominal pain	−6.3%	−23.5%	3.0%	−4.7%	−6.0%	5.3%
Headache	−7.5%	19.3%	4.1%	0.1%	5.8%	−4.2%
Nausea	8.3%	−28.9%	−0.1%	−0.7%	−4.6%	0.5%
Back pain	2.9%	−20.5%	2.3%	−0.8%	0.0%	−0.7%
Fatigue	540.1%	89.3%	2.0%	0.6%	10.4%	0.9%
Cough	−28.0%	199.0%	−15.9%	−3.5%	61.5%	−21.9%
Dizziness	24.8%	−34.7%	11.8%	−6.1%	−3.1%	−3.1%
Pharyngeal pain	73.8%	158.1%	0.4%	−4.8%	60.9%	−25.1%
Nasal congestion	13.0%	102.5%	38.8%	0.5%	51.9%	−33.9%
Vomiting	4.7%	−26.9%	−2.8%	11.7%	8.4%	8.1%
Diarrhea	0.8%	−25.0%	8.7%	−0.7%	12.9%	−7.8%
Dyspnea	−15.8%	38.0%	1.5%	−18.0%	7.0%	−9.7%
Chest pain	−27.8%	90.1%	−3.2%	−10.9%	11.7%	−4.1%
Fever	108.0%	0.6%	−8.0%	0.6%	27.2%	−0.6%
Joint pain	−4.4%	10.4%	4.6%	−1.7%	−7.2%	6.4%
Nasal catarrh	−12.1%	272.2%	10.7%	9.4%	68.1%	−28.0%
Edema	−37.8%	29.1%	−12.3%	19.8%	−24.6%	1.0%
Abnormal vaginal discharge	67.7%	−33.5%	−5.3%	−6.1%	−14.3%	20.5%
Dermatological changes	−16.3%	15.1%	25.9%	21.4%	−21.1%	11.2%
Bloating	−1.2%	−31.5%	2.6%	−11.3%	−16.5%	15.5%

Note: Q refers to a quarter of the year.

**Figure 6:**
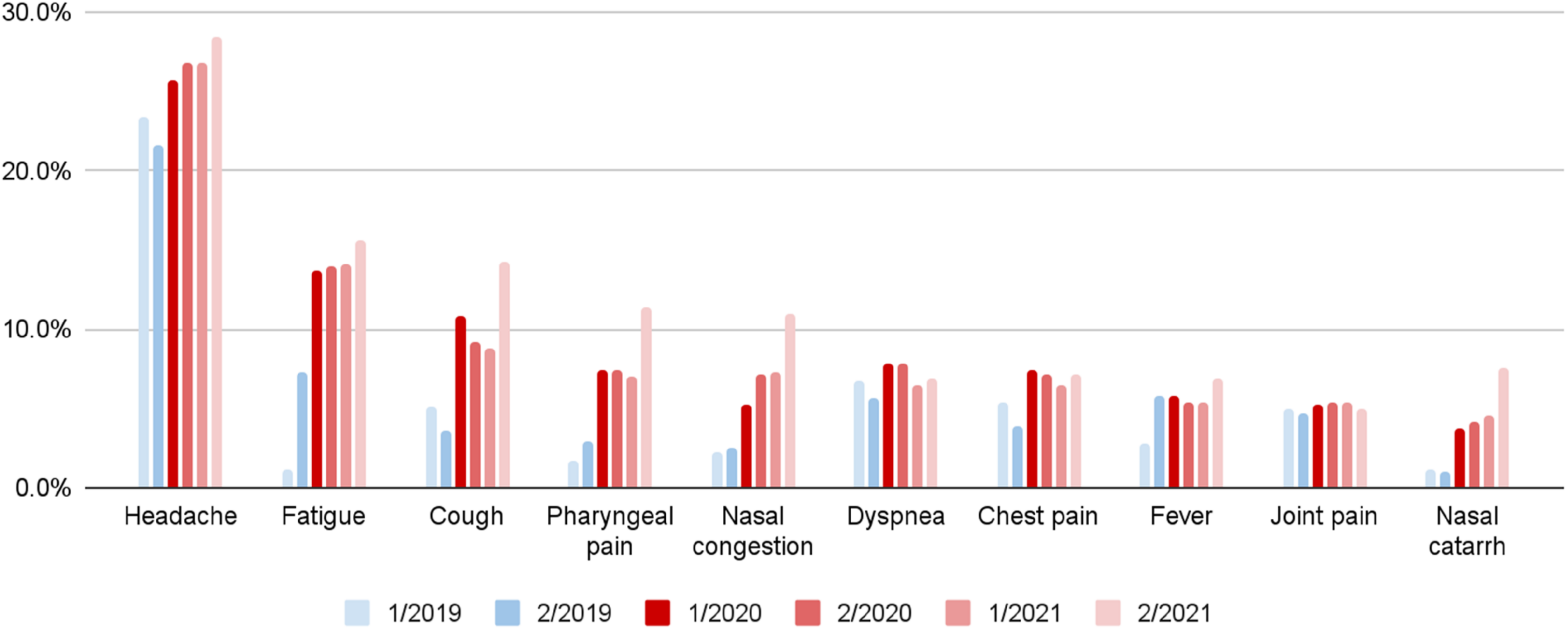
Increased symptom prevalence among pregnant patient-users by six month period (quarter 1-2/2020 vs. quarter 3-4/2019).

**Figure 7:**
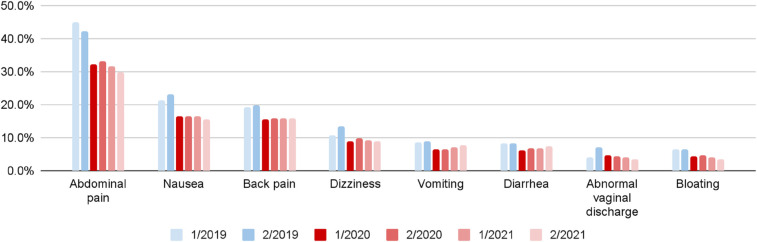
Decreased symptom prevalence among pregnant patient-users by six month period (quarter 1-2/2020 vs. quarter 3-4/2019).

Figures 6, 7 present prevalence changes across analyzed periods for the most common initial symptoms. Figure 6 focuses on symptoms with increased prevalence between the second half of 2019 and first half of 2020, while Figure 7 shows those with decreased prevalence. The greatest differences in prevalence of reported initial symptoms were observed comparing the second half of 2019 and first half of 2020. In the first half of 2020, a significant drop occurred in patient-users seeking advice about symptoms such as dizziness (−34.7%), abnormal vaginal discharge (−33.5%), bloating (−31.5%), nausea (−28.9%), vomiting (−26.9%), diarrhea (−25.0%), abdominal pain (−23.5%), and back pain (−20.5%), symptoms more typically associated with early pregnancy. In contrast, there was increased reporting of nasal catarrh (272.2%), cough (199.0%), pharyngeal pain (158.1%), nasal congestion (102.5%), chest pain (90.1%), fatigue (89.3%), dyspnea (38.0%), edema (29.1%), symptoms associated with SARS-CoV-2 infection and pneumonia.

To further explore the identified trends in initial symptoms, the data was segmented by age group by examining the volume of triage complaints among women aged 18–29 and 30–44, two groups with the greatest number of pregnant users (36,693 users in total).

**Figure 8:**
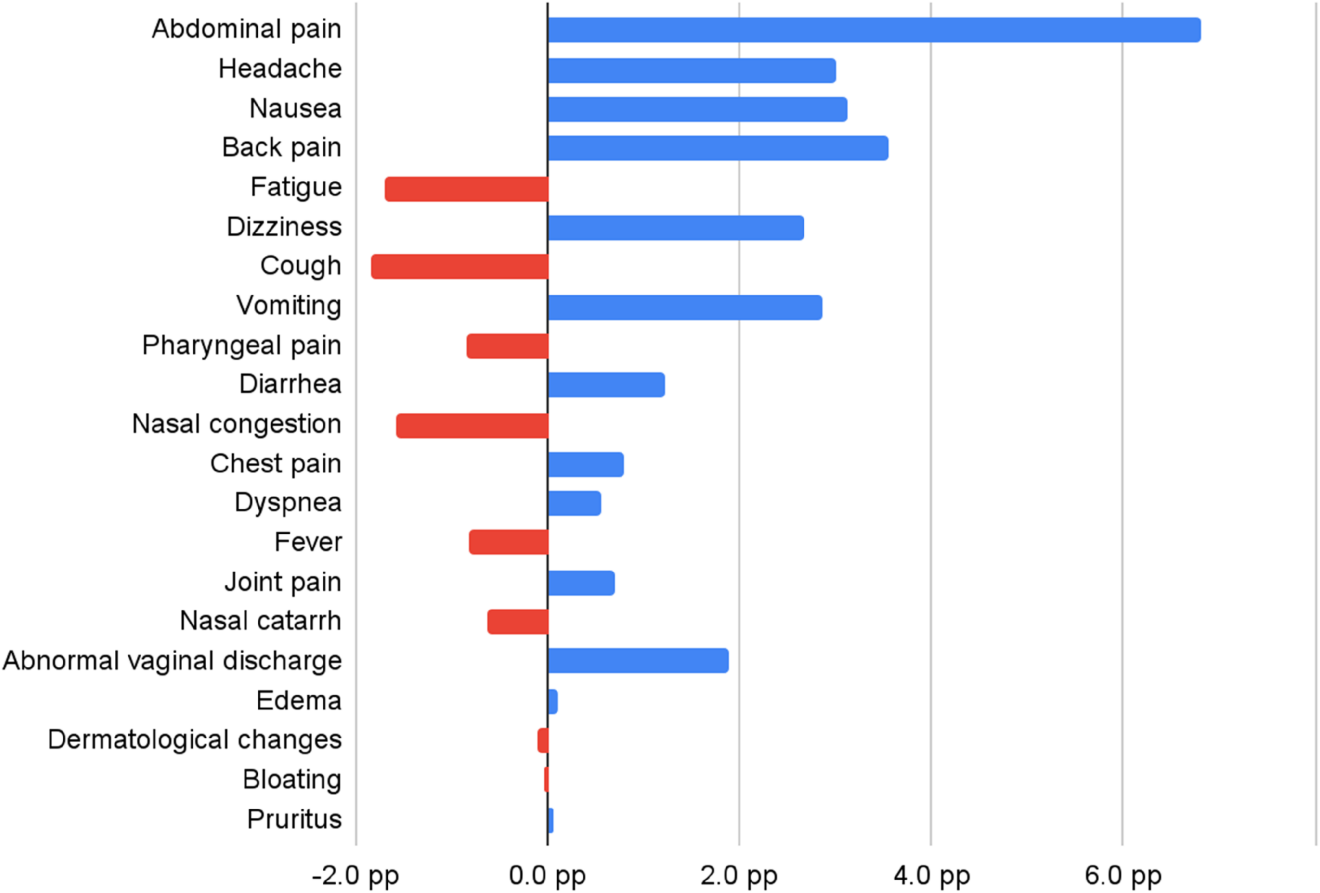
Most common initial complaints reported by pregnant users (percent point difference between 18 and 29 and 30-44 years old). A blue bar represents a symptom that was reported in higher prevalence in the 18–29 year old group and red bar represents a symptom reported more in the 30–34 year old group.

**Figure 9:**
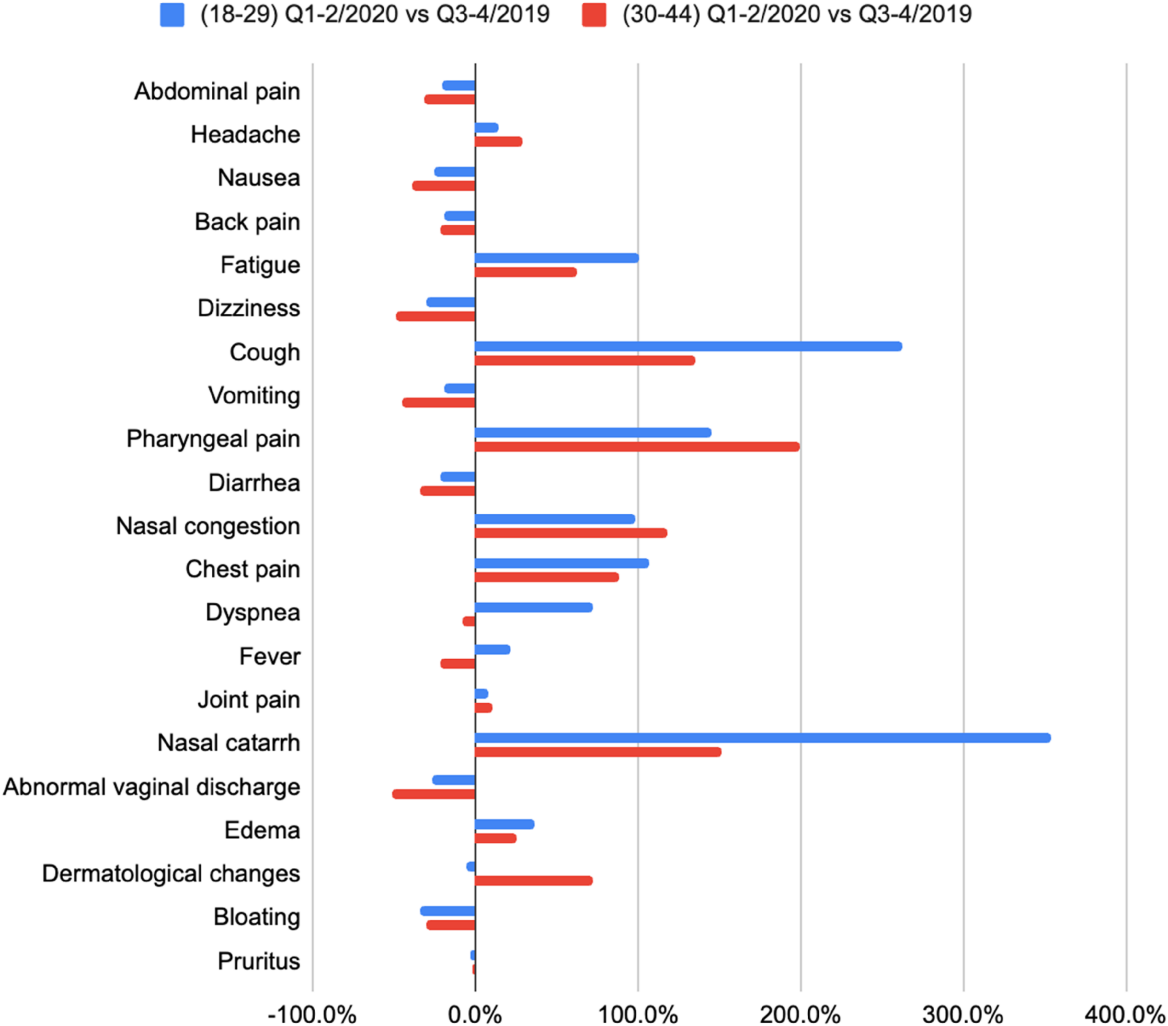
Symptom prevalence changes among pregnant patient-user in quarter 1-2/2020 vs. quarter 3-4/2019 by age group.

Table 6 presents initial complaints and episodes by age group (18–29 and 30–44), including percentage point differences, focusing on the top 21 initial complaints within each age group. Graphic presentations of these findings are found in Figures 8, 9.

**Table 6:** Most common initial complaints reported by pregnant virtual triage users 18–44 years Old.

Symptom	(18–29) Number of triage complaints	(18–29) Triage episodes with pregnant users	(30–44) Number of triage complaints	(30–44) Triage episodes with pregnant users	Percent point difference between 18 and 29 and 30–44
Abdominal pain	8,553	34.3%	3,224	27.5%	6.8 pp
Headache	6,968	27.9%	2,924	24.9%	3.0 pp
Nausea	4,360	17.5%	1,683	14.3%	3.1 pp
Back pain	4,303	17.2%	1,606	13.7%	3.6 pp
Fatigue	3,427	13.7%	1,812	15.4%	−1.7 pp
Dizziness	2,532	10.1%	877	7.5%	2.7 pp
Cough	2,517	10.1%	1,401	11.9%	−1.9 pp
Vomiting	2,093	8.4%	648	5.5%	2.9 pp
Pharyngeal pain	1,986	8.0%	1,033	8.8%	−0.8 pp
Diarrhea	1,850	7.4%	727	6.2%	1.2 pp
Nasal congestion	1,788	7.2%	1,027	8.8%	−1.6 pp
Chest pain	1,760	7.1%	735	6.3%	0.8 pp
Dyspnea	1,748	7.0%	758	6.5%	0.5 pp
Fever	1,444	5.8%	775	6.6%	−0.8 pp
Joint pain	1,354	5.4%	555	4.7%	0.7 pp
Nasal catarrh	1,228	4.9%	653	5.6%	−0.6 pp
Abnormal vaginal discharge	1,203	4.8%	345	2.9%	1.9 pp
Edema	1,121	4.5%	514	4.4%	0.1 pp
Dermatological changes	1,037	4.2%	500	4.3%	−0.1 pp
Bloating	1,018	4.1%	484	4.1%	0.0 pp
Pruritus	891	3.6%	412	3.5%	0.1 pp

Figure 8 visualizes prevalence difference across two key patient user-age groups, 18–29 years and 30–34 years old. For example, the prevalence difference of abdominal pain is equal to 6.8 pp, calculated as a difference between 34.3% (for 18–29 years old), and 27.5% (for 30–44). Symptoms where prevalence was higher among younger users were marked with blue color.

Symptoms reported more frequently by 18–29-year-old pregnant users include abdominal pain (prevalence was 6.8 pp higher), headache (3.0 pp), nausea (3.1 pp), back pain (3.6 pp), dizziness (2.7 pp), and vomiting (2.9 pp). Symptoms such as fatigue (1.7 pp), cough (1.9 pp), and nasal congestion (1.6 pp) were reported more frequently among pregnant users 30–44 years old.

Table 7 compares the prevalence of symptoms across periods Quarter 1-2/2020 and Quarter 3-4/2019 separately for each age group. Increases in prevalence of greater than 20.0% are noted in green and decreases of greater than 20.0% are red.

**Table 7:** Symptom prevalence changes among pregnant patient-users in quarter 1-2/2020 vs. quarter 3-4/2019 by age group.

Symptom	(18–29) Q1–2/2020 vs. Q3-4/2019	(30–44) Q1-2/2020 vs. Q3-4/2019
Abdominal pain	−20.4%	−31.7%
Headache	14.5%	29.4%
Nausea	−25.1%	−39.4%
Back pain	−19.0%	−21.9%
Fatigue	100.9%	62.5%
Dizziness	−30.2%	−48.4%
Cough	262.5%	135.7%
Vomiting	−19.2%	−45.6%
Pharyngeal pain	144.7%	200.0%
Diarrhea	−21.7%	−33.9%
Nasal congestion	98.3%	118.1%
Chest pain	106.5%	88.3%
Dyspnea	71.9%	−8.0%
Fever	22.3%	−20.9%
Joint pain	8.6%	10.0%
Nasal catarrh	354.1%	151.8%
Abnormal vaginal discharge	−26.5%	−51.8%
Edema	36.0%	24.8%
Dermatological changes	−4.9%	72.4%
Bloating	−34.1%	−30.1%
Pruritus	−2.5%	−1.6%

Eighteen of 21 initial complaints increased in the first half of 2020: cough and nasal catarrh prevalence increased substantially in pregnant users between 18 and 29 years old (cough by 262.5% compared to 135.7% for users 30–44, and nasal catarrh by 354.1% compared to 151.8%). Prevalence changes of vomiting, headache, and abnormal vaginal discharge were greater among those 30–44 (vomiting decreased by 45.6% compared to 30.2% in 18–29, and headache increased by 29.4% compared to 14.5%).

As shown in Figure 9, two symptoms were reported more frequently in the first half of 2020 by pregnant users 18–29 years old, and less frequently among 30–44 years old. These include dyspnea (an increase of 71.9% among users 18–29 and a decrease of 8.0% among those 30–44 years old), and fever (an increase by 22.3% in group 18–29, and a decrease by 20.9% in group 30–44). This result contradicts other known studies about the age distribution of COVID-19 symptoms (19), and requires further research to understand the factors influencing such an increase in severe COVID-19 symptoms reported by younger patient-users.

## Discussion

This analysis demonstrates that during the COVID-19 pandemic between 2019 and 2021, overall use of digital triage tools increased considerably. This trend was especially apparent during the first half of 2020, when pandemic-related infection and disease control measures were first implemented. Similar increases in the usage of telemedicine and telehealth technology has been described during and following the pandemic (4). Research has shown that symptom checkers can capture COVID-19 case trends on a national level, serving as a valuable tool for the self-reporting during pandemics and outbreaks (20). However, a significant decline in virtual triage usage following the initial pandemic surge of infection did not occur, and high triage use was sustained. Furthermore, among pregnant patient-users, we observed continuing increased usage through the end of 2021 with only a slight decline in 2022. Herling et al. reported a similarly elevated level of patient acceptance of telemedicine in gynecology services in Germany (21), however no other reports describe the adoption of digital health applications by pregnant patients during the COVID-19 pandemic. Further analysis shows the age group reporting pregnancy most often were between 18 and 44 years, where the overall population increase in VT usage was observed, aligning with US census data (22).

The most frequent complaints reported by patients in the early study period were abdominal pain, headache, back pain, nausea, vomiting and fatigue, which are the most commonly reported acute medical concerns during pregnancy (23, 24), thus VT use reflects typical low-acuity pregnancy-related clinical issues. The prevalence of many of these complaints decreased over the course of the pandemic, and other symptoms were reported more frequently. During the first half of 2020, the reported prevalence of symptoms such as nasal catarrh, cough, pharyngeal pain, nasal congestion, chest pain, fatigue, and dyspnea increased, which parallels other studies where these COVID-19 symptoms were common among pregnant patients (25, 26). A notable difference was the prevalence of fever among 32% of pregnant patients with confirmed COVID-19 infection reported by Zambrano et al. (25), higher than found in the present study (−6.0%). Also, whereas Zambrano et al. observed chest pain present in only 3.5% of pregnant patients with confirmed COVID-19 infection, we observed 6.8% of patients with this symptom in 2020. These differences may result if patients’ report VT symptoms that are more severe or distressing.

The age division of reported initial symptoms, indicates that young adults (16–19, 22–29) tend to use symptoms checkers to evaluate more severe symptoms than older patients, including symptoms of both pregnancy complications (abdominal pain, vomiting, abnormal vaginal discharge) as well as other severe symptoms that may indicate COVID-19 infection or other pulmonary disease (fever, chest pain, dyspnea). This may suggest higher trust among young adults of VT technology (28) compared to older patients who consult VT more for minor symptoms (cough, fatigue, nasal congestion). Still, regardless of age group, a considerable increase in reporting of upper respiratory symptoms (cough, pharyngeal pain, nasal catarrh, nasal congestion) occurred in all age groups, indicating higher incidence of those symptoms or increased importance among patients seeking help online during the pandemic.

Future research should follow a cohort of patients through their engagement of healthcare following VT in order to determine if the technology has any impact on maternal or neonatal health outcomes. Unfortunately, the design and data gathered for the present study does not enable such assessment. It has been demonstrated, however, that VT use can favorably change patient-user care seeking behavior when the patient had an initial care intent that was not aligned with the recommendation of VT (30, 31). In addition, in the coming years it will be critical to explore the integration of VT with other digital health tools, such as wearable devices and mobile health applications, which can broaden and deepen the individual patient-user clinical data which the AI within VT uses in formulating assessments and care recommendations.

## Conclusions

The onset of the COVID-19 pandemic at the beginning of 2020 produced a substantial increase in the number of patients seeking health information beyond standard face-to-face clinical settings, including virtual triage (21, 27, 32). Among patients using digital health tools such as VT, the number of pregnant patient-users increased usage relative to non-pregnant users. The number of pregnant patient-users seeking help through VT peaked after the initial phases of the pandemic, and did not decline even after routine access to in-person healthcare services was restored. This suggests that early adopters of VT technology found value in such digital tools, particularly among those seeking information about their pregnancy related health concerns, also indicated by research on patient satisfaction after using Symptomate, where 80.1% of all users stated that they were likely or highly likely to use VT again (28).

Initial complaints reported by pregnant patients changed over the course of the pandemic. As the pandemic progressed, a majority of pregnant patients increasingly sought information about symptoms typical of SARS-CoV-2 infection, as pregnancy related symptoms were displaced and waned. This may reflect an increased rate of COVID-19 infections among pregnant patient-users, but also likely results from the anxiety of pregnant patient-users during the pandemic, especially when pandemic disease control measures limited access to in-person primary and obstetric care, and when surges in the volume of hospitalized patients overwhelmed many national healthcare systems (1, 29).

Virtual triage demonstrates promise for sustained, stable adoption and utilization among pregnant patient-users, and its integration into the obstetrical care of pregnant patients should be the subject of continued innovation and systematic impact evaluation. Increased use of virtual health solutions by expecting mothers in order to explore and learn about typical pregnancy symptoms and complications, as well as symptoms related to SARS-CoV-2 infection, suggest that pregnant patient-users find value in online virtual learning about potential causes of their symptoms and the most appropriate care to be engaged. Further research should seek to increase understanding of how virtual triage can best allay inappropriate fears generated by routine but relatively harmless clinical issues, while also heightening early detection and appropriate care referral for urgent care when needed.

## Data Availability

The raw data supporting the conclusions of this article will be made available by the authors, without undue reservation.
